# Seaweed Extracts: A Promising Source of Antibiofilm Agents with Distinct Mechanisms of Action against *Pseudomonas aeruginosa*

**DOI:** 10.3390/md20020092

**Published:** 2022-01-21

**Authors:** Maya Rima, Jeanne Trognon, Laure Latapie, Asma Chbani, Christine Roques, Fatima El Garah

**Affiliations:** 1 Laboratoire de Génie Chimique, Université de Toulouse, CNRS, INPT, UPS, 31062 Toulouse, France; maya.rima@univ-tlse3.fr (M.R.); jeanne.trognon@univ-tlse3.fr (J.T.); latapie@chimie.ups-tlse.fr (L.L.); 2 Laboratory of Applied Biotechnology, AZM Center for Research in Biotechnology and Its Applications, Doctoral School of Science and Technology, Lebanese University, El Mittein Street, Tripoli 1300, Lebanon; asmashbani61@gmail.com; 3 Faculty of Public Health III, Lebanese University, Tripoli 1300, Lebanon; 4 Bacteriology-Hygiene Department, Centre Hospitalier Universitaire, Hôpital Purpan, 31300 Toulouse, France

**Keywords:** seaweed extracts, *Ulva lactuca*, anti-biofilm, *Pseudomonas aeruginosa*, synergistic activity, biofilm-matrix

## Abstract

The organization of bacteria in biofilms is one of the adaptive resistance mechanisms providing increased protection against conventional treatments. Thus, the search for new antibiofilm agents for medical purposes, especially of natural origin, is currently the object of much attention. The objective of the study presented here was to explore the potential of extracts derived from three seaweeds: the green *Ulva lactuca*, the brown *Stypocaulon scoparium*, and the red *Pterocladiella capillacea*, in terms of their antibiofilm activity against *P. aeruginosa*. After preparation of extracts by successive maceration in various solvents, their antibiofilm activity was evaluated on biofilm formation and on mature biofilms. Their inhibition and eradication abilities were determined using two complementary methods: crystal violet staining and quantification of adherent bacteria. The effect of active extracts on biofilm morphology was also investigated by epifluorescence microscopy. Results revealed a promising antibiofilm activity of two extracts (cyclohexane and ethyl acetate) derived from the green alga by exhibiting a distinct mechanism of action, which was supported by microscopic analyses. The ethyl acetate extract was further explored for its interaction with tobramycin and colistin. Interestingly, this extract showed a promising synergistic effect with tobramycin. First analyses of the chemical composition of extracts by GC–MS allowed for the identification of several molecules. Their implication in the interesting antibiofilm activity is discussed. These findings suggest the ability of the green alga *U. lactuca* to offer a promising source of bioactive candidates that could have both a preventive and a curative effect in the treatment of biofilms.

## 1. Introduction

Although the discovery of antibiotics has revolutionized modern medicine and has saved the lives of millions of patients, their massive use has contributed to a selection pressure on bacteria leading to the rapid emergence of multidrug-resistant strains (MDR) [1]. Unfortunately, the World Health Organization (WHO) has warned of a “post-antibiotic” world in which a supposedly life-saving drug will lose its effectiveness [2].

*Pseudomonas aeruginosa*, an opportunistic human pathogen often associated with chronic and nosocomial infections, is one of the three bacteria (*Acinetobacter baumannii* and Enterobacteriaceae) classified by the WHO as a critical priority in the search for new therapeutic strategies, due to its phenotypic and genotypic resistance towards most conventional antibiotics [3]. This ubiquitous Gram-negative bacterium is characterized by its versatile metabolic capacity, which allows it to adapt and colonize, as biofilms, different biotic and abiotic surfaces [4].

Biofilms are defined as organized populations of microorganisms adhering to each other and to a surface, enclosed in a matrix consisting of highly hydrated extracellular polymeric substances (EPS), essentially composed of exopolysaccharides, proteins, nucleic acids, and minerals [5]. This matrix, also known as the “House of Biofilm Cells”, represents up to 90% of total biofilm biomass and its value is reflected in its structural, as well as in its functional benefits to the biofilm [6]. In addition to its essential role in maintaining the architecture, stability, and growth of the biofilm, EPS ensures an “innate” tolerance by forming a mechanical barrier against the penetration of antimicrobial agents and host immune system components [7,8]. At the same time, transfer limitation participates in drastic modifications of the cellular physiology. According to the National Institutes of Health (NIH), bacterial biofilms are implicated in 65% of microbial diseases and 80% of chronic infections [9].

*P. aeruginosa* biofilm presents the hallmark of long-term infection persistence and progression from colonization to infection that can lead to death, particularly in immunocompromised subjects and in cystic fibrosis (CF) patients [4]. In addition to its intrinsic and acquired resistance, the extraordinary ability of this bacterium to form biofilm accentuates its strength by providing a protective barrier against host defenses, as well as against anti-*Pseudomonas* antibiotics [10].

For all these reasons, the search for approaches to effectively prevent or treat biofilm-associated infections is currently the focus of great interest. However, despite the protective effect bestowed on bacterial cells in the biofilm state, an important feature to be considered is the total reversibility of the specific resistance when biofilms are disrupted, leading the phenomenon to be considered as a transitory loss of susceptibility rather than true resistance [8,9]. This definitely encourages the search for new strategies to inhibit biofilm formation and disrupt existing biofilms.

In this context, natural compounds can be a boon for the discovery of novel bioactive agents, including biofilm inhibitors [11]. In particular, the capacity of marine organisms to overcome stressful environmental conditions and their ability to protect themselves from bacterial invasion suggest their great richness in bioactive compounds [12]. Macroalgae, which are traditionally used for both nutritional and medicinal purposes, offer a valuable source of bioactive molecules with a wide spectrum of biological activities (anti-inflammatory, antitumor, antiviral, antimicrobial, neuroprotective, etc.), proved both in vitro and in vivo [13,14]. The availability of algal resources and the diversity of their chemical composition within green (Chlorophyta), red (Rhodophyta), and brown (Phaeophyta) algae, point to their huge potential for industrial applications [15,16].

Interestingly, a halogenated furanone isolated from the red alga *Delisea pulchra*, endemic to the south-eastern coast of Australia, was the first molecule identified as having an inhibitory activity on the bacterial communication system known as quorum sensing (QS), a mechanism essential to biofilm formation [17]. In particular, this natural molecule has been demonstrated to interfere with the *N*-acylhomoserine lactone (AHL) based quorum sensing regulatory systems of several Gram-negative bacteria [1] and several studies have proved the interest of using natural products as sources of QS inhibitors [18,19].

The present study aims to explore the potential of three seaweed species as sources of antibiofilm agents against *P. aeruginosa*. The green (*Ulva lactuca* “Sea lettuce”), the brown (*Stypocaulon scoparium* “Sea broom”), and the red (*Pterocladiella capillacea*) algae were chosen for their wide range of demonstrated bioactivities [12,14,20]. The originality of this study lies in the fact that algae are scarcely explored for their potential antibiofilm activity [21,22,23,24]. After preparation of different extracts, their antibiofilm activity was evaluated using two complementary assays: the crystal violet staining method and the quantification of adhered living cells by the colony-forming unit (CFU) counting method Both effects on the initial adhesion and biofilm progression and on 24-h-old biofilms were evaluated. Fluorescence microscopy observation was combined in order to confirm our results and to demonstrate a potential modification of the biofilm morphology. Finally, the potential synergistic antibiofilm activity between the most active extract and tobramycin and colistin, two antibiotics which are generally used to combat *P. aeruginosa* lung infections, was analyzed [25].

## 2. Results

### 2.1. Extraction Yields of Different Seaweed Extracts

Seaweed extracts were prepared by successive maceration in different solvents with increasing polarity, with cyclohexane as the least polar solvent used (P’: 0.2) and methanol the most polar one (P’: 5.1). As expected, the yields of seaweed extracts were affected by the polarity of the extraction solvent used (Table 1). In fact, for the three algae evaluated in this study, the highest extraction yield was recorded for the methanolic extracts, resulting in 12.1, 1.4, and 7.3% (*w*/*w*) for green, brown, and red seaweed, respectively. Moreover, the number of extraction repetitions required with methanol to achieve a complete extraction demonstrates the richness of these algae in polar compounds in comparison with their content in non-polar ones.

### 2.2. Assessment of the Inhibitory Effect of Extract on BIOFILM formation—Extracts Added at t_0_

#### 2.2.1. Screening of Algal Extracts for Their Inhibitory Effect on PAO1 BIOFILM Formation and Growth—Crystal Violet (CV) Staining Method

**Figure 1:**
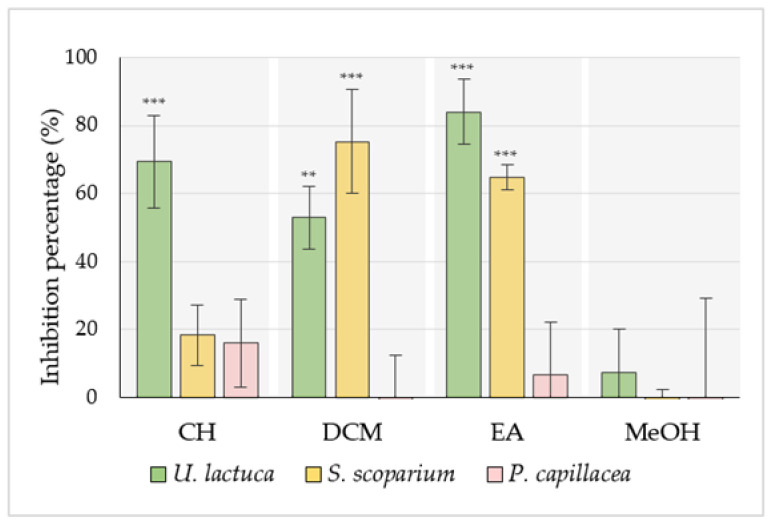
Effect of different algal extracts (50.0 µg/mL) on PAO1 biofilm formation, assessed using the CV staining method. Extracts were added at t_0_ to evaluate their effect on biofilm formation and growth. Results are expressed as the inhibition percentage (IP_CV_ %) mean ± SD, from three independent experiments. CH, DCM, EA, and MeOH are cyclohexane, dichloromethane, ethyl acetate, and methanol extracts, respectively. Statistically significant difference (**, *p*-value < 0.01, ***, *p*-value < 0.001) between the extract and the related untreated control is indicated.

The initial screening was carried out by the crystal violet staining method, which allowed the entire biomass of the biofilm to be quantified. Note that all antibiofilm assays were conducted in the minimum modified biofilm broth (MBB), which promotes the formation of the biofilm, rather than planktonic growth, by creating stressful conditions [26]. This was confirmed by comparing the PAO1 growth curve in this medium with growth in the rich MHB medium (Supplementary Materials Figure S2). In order to evaluate their effect on the first stage of bacterial biofilm formation (from adhesion to proliferation under adherent status), algal extracts (50.0 µg/mL) were first added at t_0_. Their ability to reduce the biofilm biomass is compared to the control and expressed as inhibition percentages (IP_CV_) in Figure 1.

Concerning CH extracts, only the one derived from the green alga was able to significantly reduce PAO1 biofilm biomass (IP_CV_ = 69.4 ± 13.6%) (***, *p*-value < 0.001). On the other hand, DCM extracts obtained from both green and brown algae exhibited considerable antibiofilm activity leading to biomass reductions of 52.9 ± 9.2% (**, *p*-value < 0.01) and 75.2 ± 15.4% (***, *p*-value < 0.001), respectively. Regarding EA extracts, results showed that the one derived from the green alga had the best ability to reduce PAO1 biofilm biomass (IP_CV_ = 84.0 ± 9.6%) (***, *p*-value < 0.001). EA extract obtained from the brown alga also presented a notable activity (IP_CV_ = 64.8 ± 9.2%) (***, *p*-value < 0.001). Note that no significant activity was recorded for any MeOH extracts or red alga *P. capillacea* extracts.

#### 2.2.2. Effect of Selected Active Extracts on the Number of Adhered Bacteria—CFU Counts Method

Following the screening by the CV method, extracts with an IP_CV_ higher than 50% were selected for evaluation by the CFU counting method of their effect on adhered cells. Results obtained by the CFU counting method and by the CV staining method (already displayed in Figure 1) are presented in Table 2 in order to compare them and thus search for a potential correlation. Results showed that the CH extract of the green alga *U. lactuca* was the only one to show a significant inhibitory activity (**, *p*-value < 0.01), leading to 5.9 ± 0.1 log CFU/mL versus 6.4 ± 0.2 log CFU/mL in the related untreated control (0.5 ± 0.1 log reduction). While the activity of the EA extract derived from the green alga was only demonstrated by the CV staining method, a consistency between the two methods (IP_CV_ = 69.4 ± 13.6%; IP_CFU_ = 67.2 ± 17.2%) was observed for the CH extract. This can be explained by two different modes of antibiofilm action for these extracts.

In order to confirm this finding, the PAO1 biofilms treated with these two active extracts were analyzed by epifluorescence microscopy.

#### 2.2.3. Phenotypic Observations of Biofilms by Epifluorescence Microscopy

**Figure 2:**
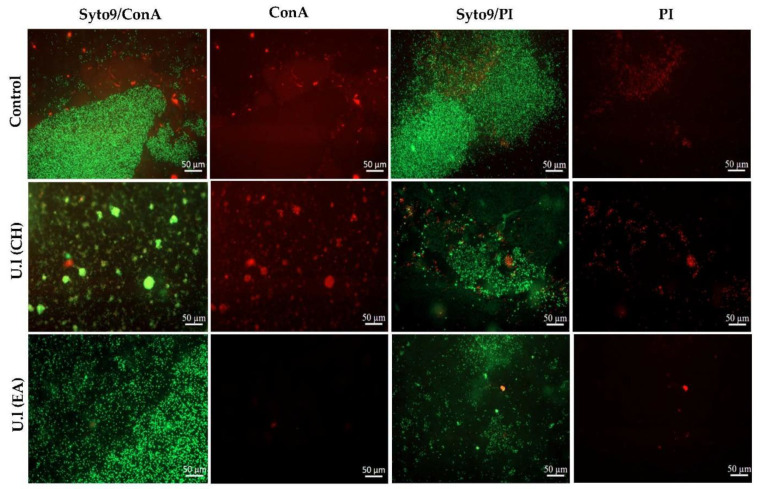
Epifluorescence microscopy images of PAO1 biofilms incubated in MBB medium at 37 °C for 24 h without extract (control) or with one of the two active extracts (cyclohexane or ethyl acetate extract) of the green alga *U. lactuca* at 50.0 μg/mL. Extracts were added at t_0_. Biofilms were stained with Syto9 for cells (green-fluorescent), with concanavalin A for the matrix sugars (red-fluorescent), and with Syto9 and propidium iodide (PI) to differentiate live and damaged cells, respectively. U.l (CH) and U.l (EA) are cyclohexane and ethyl acetate extract, respectively, derived from the green alga *U. lactuca*. (Magnification: ×20).

For the CH and EA extracts originating from the green alga, *U. lactuca*, the effect on the biofilm structure and composition was examined by epifluorescence microscopy, by labeling (i) cells and matrix sugars and (ii) live/damaged cells (Figure 2). The phenotype of the biofilm was displayed after 24 h of incubation in MBB medium, with or without extract.

Compared to the typical control biofilm consisting of bacterial cells surrounded by a well-distributed matrix, biofilms grown in the presence of extracts showed dissimilar structures. A decrease in cell number was confirmed when the CH extract was added at t_0_, with characteristic separated bacterial aggregates encased in an associated matrix (conA staining). Damaged cells or eDNA are also more likely to be in the form of aggregates than in isolation. On the other hand, a potential effect on the matrix was demonstrated in the biofilm treated with the EA extract, leading to scattered adherent cells lacking matrix (conA staining). Furthermore, the differentiation between living and damaged cells by Syto9/PI revealed the prevalence of living cells.

### 2.3. Effect of Selected Algal Extracts on PAO1 24 h-Old Biofilm—Extracts Added at 24 h

**Figure 3:**
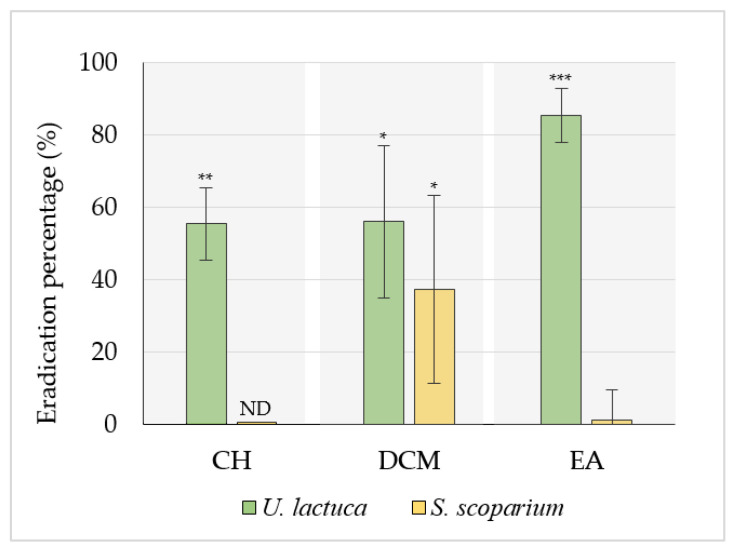
Effect of selected algal extracts (50.0 µg/mL) on PAO1 24 h-old biofilm assessed using the CV staining method. Extracts were added at t_24h_ to evaluate their effect on 24 h-old biofilms. Results are expressed as the eradication percentage mean ± SD from three independent experiments. Statistically significant difference (*, *p*-value < 0.05, **, *p*-value < 0.01, ***, *p*-value < 0.001) between the extract and the related untreated control is indicated. ND: not determined.

The extracts selected after the first screening (IP_CV_ > 50%) were subjected to an evaluation of their ability to eradicate a 24-h-old biofilm. For this purpose, extracts were added at t_24h_, followed by overnight incubation. The biomass remaining adhered after treatment of PAO1 biofilm with extracts or not was first quantified by the CV staining method (Figure 3). Results are expressed as eradication percentages.

Results revealed that the EA extract of the green seaweed exhibited the best eradication activity (EP_CV_ = 85.6 ± 7.4%). In addition, CH and DCM extract also obtained from *U. lactuca* displayed a moderate activity on PAO1 24 h-old biofilm, leading to 55.5 ± 10.0% and 56.1 ± 21.0% of eradication, respectively.

**Figure 4:**
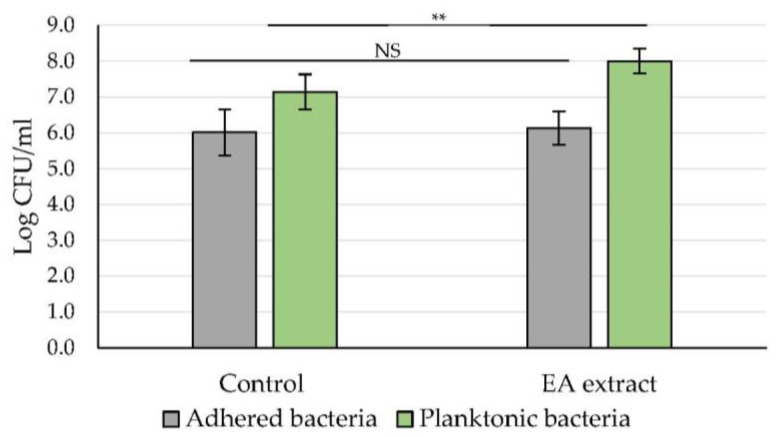
Effect of the *U. lactuca* EA extract (50.0 μg/mL) on PAO1 24 h-formed biofilm using the CFU counting assay. Both adherent and planktonic bacteria were quantified (CFU counts) after 24 h incubation in the MBB medium. Results are expressed as mean (log CFU/mL) ± SD from three independent experiments. Statistically significant difference (**, *p*-value < 0.01) between extract and control is indicated. EA: ethyl acetate extract. NS: not significant.

On the other hand, the effect of the most active extract (EA extract with EP_CV_ > 80%) was evaluated using the CFU counting assay, with quantification of both adhered and detached (planktonic) cells (Figure 4). While no notable effect was observed on adhered cell counts, a significant increase in the number of detached cells was measured (**, *p*-value < 0.01) in the presence of EA extract (8.0 ± 0.3 log CFU/mL) compared to the control (7.1 ± 0.5 log CFU/mL). In order to exclude a possible growth promoter effect of the EA extract, its effect on planktonic growth was examined by plotting the growth curve. Results validated the absence of any significant effect of the EA extract on planktonic growth (Supplementary Materials Figure S2).

### 2.4. Evaluation of the Synergistic Antibiofilm Activity of EA Extract in Combination with Tobramycin or Colistin

**Figure 5:**
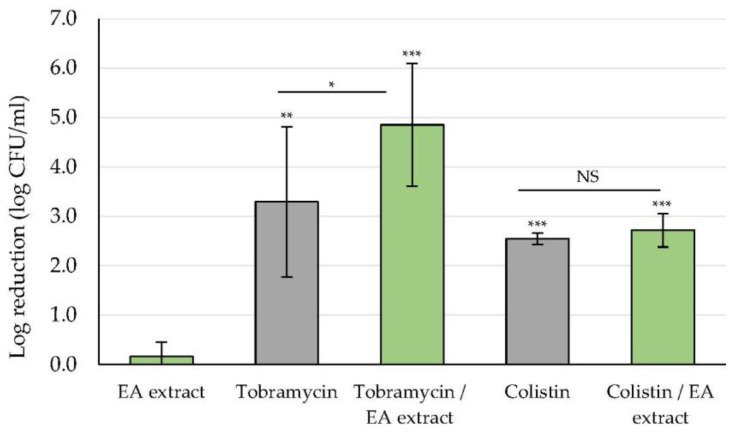
Synergistic effect of the *U. lactuca* EA extract (50.0 µg/mL) and tobramycin (2 µg/mL) and colistin (16 µg/mL) on PAO1 biofilms using the CFU counting assay method. The EA extract was added at t_0_. The EA extract/antibiotic combination was added after 24 h of incubation at 37 °C as antibiotics alone. Results are expressed as means of log reduction in comparison with the related untreated control (log reduction (log CFU/mL) ± SD) from three independent experiments. Statistically significant differences (**, *p*-value < 0.01, ***, *p*-value < 0.001) between the log CFU/mL number remaining after treatment with the EA extract/antibiotic combination or with the antibiotics alone and that in the appropriate untreated control are indicated. Statistically significant difference (*, *p*-value < 0.05) between the log CFU/mL number remaining after treatment with the EA extract/antibiotic combination vs. antibiotic alone. NS: not significant.

Since the CV staining method showed EA extract originating from the green alga *U. lactuca* to be the most effective in reducing the formation of the biofilm, as well as in eradicating previously formed biofilms, the potential synergy of the extract with two conventional antibiotics was evaluated following the CFU counting method. The choice of this evaluation method was based on the demonstrated responsiveness of CFU counting method in treatment efficacy testing in comparison with the CV staining method [27]. For comparison, the antibiofilm activity of tobramycin and colistin alone was evaluated on 24 h-old untreated biofilms, while the effect of the antibiotic/EA extract combination was determined on 24 h-old biofilms, previously exposed to the EA extract for 24 h (Figure 5). Results confirmed that EA extract had no significant effect on adherent CFU counts, while tobramycin and colistin were able to induce a 3- or 2-log significant reduction, respectively. The potential synergy was expressed by comparing the logarithmic reduction in biofilm treated with each antibiotic alone with that in biofilm treated with the corresponding EA extract/antibiotic combination. Results showed that the logarithmic reduction relative to the corresponding untreated control was statistically higher after treatment with the tobramycin/EA extract combination (4.9 ± 1.2 CFU/mL of log reduction) than that obtained with tobramycin treatment alone (3.3 ± 1.5 CFU/mL of log reduction). In contrast, no significant synergy was observed between the EA extract and colistin.

### 2.5. Analysis of the Chemical Composition of Extracts by GC–MS

In an attempt to identify molecule(s) responsible for the demonstrated antibiofilm activity of the two selected active extracts, an analysis of the chemical composition of extracts was carried out by GC–MS (Table 3, Supplementary Materials Figure S5). Among the identified molecules, we found three phenolic compounds: 2,4-di-tert-butylphenol, 2,6-bis(1,1-dimethylethyl)-4-(1-methyl-1-phenylethyl)-phenol, and 2,4-Bis(dimethyl benzyl)-6-t-butylphenol. However, these compounds were also detected in inactive extracts.

## 3. Discussion

At this critical time when pathogens’ development of multiple resistance pathways has enabled them to outgrow our ability to effectively control them, we find ourselves facing a serious public health problem, since most conventional antimicrobial agents are no longer functional. In this context, the marine world, a habitat of immense biodiversity, offers a source of inspiration in the search for natural alternatives with novel mechanisms to prevent and/or treat life-threatening diseases [16]. Despite the richness of seawater in bacteria (≈1 million cells/mL of seawater), such as *Pseudomonas* species, and the correspondingly high risk of colonization by a bacterial biofilm, many marine organisms, particularly sessile ones such as algae, successfully control this bacterial threat, which suggests their innate ability to synthesize metabolites to protect themselves [28]. Several studies are emerging, bringing evidence of the significant antimicrobial, antibiofilm, and antifouling activities of extracts and compounds derived from green, brown, and red macroalgae [12,20].

In the present study, the extracts of three macroalgae were explored for their potential antibiofilm activity against the “superbug” *P. aeruginosa*. Great interest has been focused on the search for synthetic and natural alternatives to conventional antibiotics to overcome the strong ability of this pathogen to form deleterious biofilms that override antibiotherapy [29]. In this context, the present study focuses on the screening of various seaweed extracts (mixtures of compounds) for their possible antibiofilm activity. Different approaches are combined in an attempt to determine their potential mechanisms of action and select the most promising extracts for further studies.

To explore their potential antibacterial and antibiofilm activities, different extracts were prepared from the three seaweeds examined in this study, using solvents of increasing polarity. As expected, the extraction yield depends on the polarity of the solvent used. Results showed that the dry matter of the three tested algae was richer in polar compounds than in nonpolar ones, since the highest yield of the crude extract was obtained with methanol (Table 1). These results are not unexpected, given that macroalgae are characterized by a high carbohydrate content (that can reach approximately 76% of their dry weight) versus a low lipid content [30]. Besides, this is in accordance with recent studies that have demonstrated the richness of the red alga *P. capillacea* and the green alga *U. lactuca* in polar compounds [31,32].

The first screening of extracts (50.0 µg/mL) for their ability to inhibit the formation and the development of PAO1 biofilms was performed using the CV staining method. Although this method provides a good estimate of the total biofilm biomass by marking EPS, especially the polysaccharides, it is not informative on the viability and the number of adhered cells. This makes it necessary to combine the CV assay with the more accurate CFU counting method [33]. Furthermore, Allkja et al., 2021 proved that the CFU counting assay is more responsive in treatment experiments than CV staining, due to potential interaction between the treatment and the dye [27]. Results obtained by adding extracts at t_0_ revealed that those derived from the green alga *U. lactuca*, particularly CH and EA extracts, are the most promising in reducing bacterial adherent biomass, in comparison to the two other algae tested here (Table 2). It should be noted that the potential bactericidal effect of these two selected extracts at the tested concentration (50.0 µg/mL) was checked in order to confirm that the observed effect is definitely related to an antibiofilm activity. No bactericidal effect (neither on 10^2^ nor on 10^5^ CFU/mL) was recorded for these two extracts (Supplementary Materials Table S3).

By evaluating different types of extract, the value of green alga *U. lactuca* has been highlighted by various studies revealing its richness in bioactive compounds suitable for pharmaceutical (antioxidant, anti-proliferative, etc.) cosmetic, nutritional, and energy applications [34,35,36].

To the best of our knowledge, the only publication that has evaluated the antibiofilm activity of this green alga against *P. aeruginosa* by the CV method demonstrated the ability of a MeOH extract, prepared by a single maceration in methanol, to reduce total biomass [37]. This difference with our results can be attributed to many parameters, such as the extraction method, the bacterial strain and the biofilm formation conditions. Whereas rich media are commonly used for the evaluation of antibiofilm activity, in this study, PAO1 biofilms were grown in a low-nutritive medium, and using a low inoculum concentration, in order to promote biofilm formation through the growth of adherent cells. Moreover, our extracts were tested at a rather low concentration (50.0 µg/mL) to avoid solubility issues. On the other hand, due to the high variability of protocols and conditions in biofilm experiments (environmental factors, inoculum preparation, etc.) and quantification, especially those based on spectrophotometry, such as the CV staining method; data comparison between studies is very complicated [38].

Regarding the CH extract derived from the green alga *U. lactuca*, results of CV (IP_CV_ = 69.4 ± 13.6%) and CFU (IP_CFU_ = 67.2 ± 17.2%) assays were consistent, which implies a significant effect on biofilm biomass formation and growth, as well as on the number of adhered cells (Table 2). An alteration in the morphology of the cell aggregates that formed was also revealed by microscopic analysis (Figure 2). On the other hand, when tested on a 24 h-old biofilm, the ability of CH extract to reduce biofilm biomass was moderate, which suggests an effect restricted to the early stages of biofilm formation (Figure 3). Such a mechanism of action has been observed with a synthetic compound, N-(2-pyrimidyl)butanamide (C11 compound), designed to be a structural analogue of the N-butanoyl-L-homoserine lactone (C4-AHL) [26]. AHLs are signal molecules involved in the quorum sensing (QS) cell-to-cell communication system, a key factor in virulence and in biofilm formation. This “chat circuitry” requires the production, detection, and response to signal molecules leading to the synchronization of bacterial group behavior. In *P. aeruginosa*, three major QS systems are well described: *rhl* and *las* systems based on signal molecules belonging to acyl-homoserine lactones (C_4_-HSL and C_12_-HSL) and the *pqs* system regulated by 2-alkyl-4-quinolone (AQs) molecules [39,40]. Interestingly, C11 is able to prevent *P. aeruginosa* biofilm formation and proliferation only when added during the initial stages, with a dose-dependent effect, and with demonstrated antagonistic effect of the C4-AHL [26,41].

Concerning the effect of the EA extract, also derived from the green alga *U. lactuca,* on PAO1 biofilm formation and growth, a significant activity was recorded only with the CV staining method (IP_CV_ = 84.0 ± 9.6%) (Table 2). Since the main objective of this assay is to quantify the total biofilm biomass, including EPS, these results can be explained by a potential action on the production and/or degradation of the biofilm matrix. This hypothesis is supported by the epifluorescence microscopic analysis, which proved that addition of the EA extract leads to the formation of a biofilm characterized by an undefined spread matrix (Figure 2). Interestingly, this extract also showed considerable efficiency in reducing a 24 h-old biofilm by the CV method (EP_CV_ = 85.5 ± 7.4%) (Figure 3). This finding allowed us to select the EA extract and use the CFU method to explore the effect of the extract on the number of remaining adhered cells, as well as on the number of planktonic cells released. Results showed a significant increase in planktonic cells, while no effect on the adhered cell counts was observed (Figure 4), which can be attributed to a matrix modification that promotes the release of biofilm cells. This mechanism of action targeting biofilm structure and morphology has been described for usnic acid, a secondary lichen metabolite [42]. *P. aeruginosa* biofilm grown on a usnic acid-loaded polymer formed an altered structure consisting of microcolonies separated by interstitial void areas. Furthermore, Powell et al., 2018 have demonstrated the ability of alginate oligosaccharides derived from the brown alga *Laminaria hyperborea* to decrease *P. aeruginosa* biofilm biomass by disrupting its EPS network [43]. The function of the EPS is not limited to providing a protective barrier against exogenous factors, it also ensures nutrition, hydration, and intercellular interaction within the biofilm. In this scenario, and given its major role in the formation, development, and maintenance of biofilms, the EPS matrix has become a potential target in the search for novel anti-biofilm strategies such as the use of alginate lyase, DNase, or mucolytic agents, which aim to impair the complex structure of biofilms and consequently eradicate them or reduce their high resistance to antimicrobial treatments [44].

On the other hand, several studies have focused on the search for a therapy that combines an antimicrobial agent with an innovative adjuvant, especially one that can disassemble the biofilm matrix. This can be considered as a good therapy that aims to minimize the long-term administration of high doses of antibiotics [44]. The lack of biofilm sensitivity towards antibiotics is a well-known, ubiquitous phenomenon caused by a combination of factors. Generally, a biofilm’s complexity and heterogeneity can hinder the efficiency of antibiotics by many mechanisms: (1) the restricted penetration ensured by the EPS matrix components interacting with antibiotics, (2) the physiological tolerance associated with the formation of a subpopulation within the biofilm, characterized by a slower cell metabolism, leading to the inactivity of antibiotics that target fundamental cellular processes (replication, protein or cell wall synthesis, etc.), (3) tolerance based on specific genes whose expression is strictly associated with biofilm formation [45].

Thus, the possible synergistic activity between the active EA extract, which acts by potentially affecting the PAO1 matrix structure, and tobramycin or colistin antibiotics, commonly used in the treatment of *P. aeruginosa* infections, was evaluated. Tobramycin is a polycationic aminoglycoside antibiotic with hydrophilic properties. Its antibacterial mechanism of action is based on its ability to bind to ribosomal subunits, resulting in suppression of mRNA translation and subsequently the inhibition of protein synthesis [46]. Colistin is a polypeptide antibiotic belonging to the polymyxin family, with amphiphilic and cationic properties. Its binding to LPSs and phospholipids of the outer membrane of Gram-negative bacteria leads to the disruption of the cell membrane with leakage of intracellular contents and, finally, to cell death [47].

In the present study, EA extract/antibiotic combinations were evaluated on 24 h-old biofilms exposed to the EA extract. It should be noted that the tested antibiotic concentrations were selected in a previous study based on the level reached in the serum (for tobramycin) or sputum (for colistin) 1 h after administration of a single dose, which corresponded to 8 µg/mL for tobramycin and 32 µg/mL for colistin [41]. However, since these concentrations led to a strong biofilm reduction in vitro (data not shown), they were lowered to 2 and 16 µg/mL for tobramycin and colistin, respectively, in order to detect a potential synergistic effect. Results showed a significant increase in the antibiofilm activity of tobramycin against EA-extract-pretreated biofilm (Figure 5). In contrast, no synergistic activity was recorded with colistin. This can be explained by the difference in the mechanisms involved in biofilm-associated tolerance to aminoglycoside antibiotics (tobramycin) and antimicrobial peptide (colistin) and/or by a possible denaturing effect of EA extract on colistin.

For aminoglycoside antibiotics, which act at the intracellular level by targeting bacterial protein synthesis, various studies have highlighted the major role played by negatively charged EPS matrix in limiting the diffusion of such polycationic compounds through the biofilm, thus blocking their effects. For instance, alginate, a polyanionic exopolysaccharide and a component of *P. aeruginosa* biofilm matrix, has been shown to have a crucial function in protecting the biofilm from polycationic aminoglycosides, such as tobramycin, through ionic interactions [48].

In the present study, the EA extract has been proven to significantly reduce the total biomass, potentially by altering the EPS matrix structure and architecture of *P. aeruginosa* biofilms. Thus, the synergistic effect observed with tobramycin may be explained by the partial restoration of the susceptibility of PAO1 EA extract-pretreated biofilms. The absence of total recovery of biofilm sensitivity to tobramycin may be linked to other factors related to the biofilm state itself, such as the involvement of efflux pumps (e.g., MexAB-OprM) or the modification of cellular targets [49]. Interestingly, a synergistic effect with tobramycin has also been demonstrated with the C4-HSL analogue (C11) mentioned above, and also the halogenated furanone, known as a substance antagonistic to the bacterial QS communication system [41,50], since the efficacy of tobramycin on furanone-treated *P. aeruginosa* biofilms is exerted on both the surface cells and those present in the deepest layers, while the antibiotic had a limited effect on untreated biofilms.

On the other hand, the absence of synergy between the EA extract and colistin can be explained by its lower retention by the EPS matrix, in comparison with the polycationic tobramycin. Furthermore, evidence has been provided that biofilm tolerance to antimicrobial peptides is correlated with eDNA-mediated activation of *pmr/arn* operon, encoding the LPS modification enzyme [45].

To progress towards the identification of the bioactive compounds present in the two selected extracts, an analysis of the chemical composition of all extracts was performed by GC–MS (Table 3). Various molecules have been identified, some of which have already been described for their biological activity, such as the 2,4-di-tert-butylphenol [51]. In fact, Viszwapriya et al., 2016 have demonstrated the ability of this phenolic compound to inhibit *Streptococcus pyogenes* biofilm formation along with a reduction in EPS matrix production [52]. Moreover, a synergistic antibiofilm activity of this phenol with gentamycin has been reported against *Serratia marcescens* [53]. However, since most of the identified compounds were also detected in the inactive extracts, such as the extracts derived from the red alga, their specific implication in the demonstrated antibiofilm activity of the two active extracts has to be confirmed by further purification and analyses to identify and quantify the active molecule and/or the effective mixture.

Finally, as the QS communication system is a key factor in bacterial biofilm formation, the two active extracts discovered in this study may potentially act on this complex system and/or on other factors regulated by QS, such as the production of rhamnolipids. This biosurfactant, controlled by the *rhl* QS system, is involved in the different stages of biofilm formation, particularly in the mediation of cell dispersion [54]. Thus, the present results encourage towards elucidating the potential direct and/or indirect anti-QS activity of these extracts.

## 4. Materials and Methods

### 4.1. Collection of Algal Materials

Seaweed samples belonging to three different groups (green alga *Ulva lactuca*, brown alga *Stypocaulon scoparium,* and red alga *Pterocladiella capillacea*) were manually collected in the Mediterranean Sea, from the northern Lebanese coast, particularly from El Mina in Tripoli in September 2019 (Supplementary Materials Figure S1). After collection, the fresh macroalgae were rinsed with seawater to remove impurities such as particles of adhered sand or epiphytes. The samples were immediately transported to the laboratory of applied biotechnology, AZM research center, Lebanese university, Tripoli, Lebanon, where they were rigorously washed with distilled water. Then, seaweed samples were air-dried in a dark place at room temperature (20–27 °C) for several weeks and weighed continuously until they were completely dry. The dried samples were ground into a fine powder in order to facilitate extractions, and were then transported in sealed bags to the Laboratoire de Génie Chimique of Toulouse, France, where the extractions were carried out.

### 4.2. Organic Solvents, Chemicals and Antibiotics

The solvents used in this study were cyclohexane 99.5% (Sigma–Aldrich, St. Quentin Fallavier, France), dichloromethane 100% (VWR, Rosny-sous-Bois, France), ethyl acetate 99.9% (VWR, Rosny-sous-Bois, France), methanol 99.8% (Sigma–Aldrich, St. Quentin Fallavier, France) and ethanol 96% (Sigma–Aldrich, St. Quentin Fallavier, France). Unless otherwise mentioned, all chemicals, including dyes and antibiotics, were purchased from Sigma–Aldrich, St. Quentin Fallavier, France.

### 4.3. Bacterial Strain and Culture Media

The bacterial strain used in this study was *Pseudomonas aeruginosa* PAO1 (CIP 104116), purchased from the collection of the Pasteur Institute (Paris, France) and preserved at −80 °C. The inoculum used in each experiment came from a second subculture on Trypticase soy agar (BioMérieux, Crapone, France) that was incubated under aerobic conditions at 37 °C for 24 h. A low-nutritive medium, named minimum biofilm broth (MBB) was used for the biofilm formation and the evaluation of the antibiofilm activity of extracts, in order to create stressful conditions and subsequently promote biofilm formation and growth of adherent cells rather than planktonic growth. The MBB 10X medium is composed of FeSO_4_, 7 H_2_O (0.005 g/L), Na_2_HPO_4_ (12.5 g/L), KH_2_PO_4_ (5.0 g/L), (NH_4_)_2_SO_4_ (1.0 g/L), glucose (0.5 g/L) and MgSO_4_, 7 H_2_O (0.2 g/L) [26].

### 4.4. Preparation of Seaweed Extracts

In order to extract a maximum of seaweed constituents, a successive extraction method using selective solvents with increasing polarity (cyclohexane, dichloromethane, ethyl acetate, and methanol) was adopted [55]. One hundred grams of the dried samples of each alga were macerated successively in 1 L of each solvent for 2 h under magnetic agitation. Crude extracts were recovered after filtration using a Büchner funnel followed by solvent evaporation using a rotary evaporator under vacuum at 40 °C. Note that maceration with the same solvent was repeated until discoloration of the filtrate. In this case, the different extracts obtained from the same solvent were combined.

The extraction yield was then calculated using the following formula (1), where W_2_ is the weight of the extract residue after solvent evaporation and W_1_ is the weight of the algal matrix initially used in the extraction (100.0 g).

(1) $$\mathrm{Extraction}\;\mathrm{yield}\;\left(\%\right)=\left(\frac{W_{2}}{W_{1}}\right)\times 100$$

To evaluate their bioactivity, extract solutions were prepared by dissolving the extracts in sterile distilled water (SDW) at 100.0 µg/mL, using an ultrasonic bath (VWR ultrasonic cleaning bath, 45 kHz) for 1 to 6 h until complete dissolution. Extract solutions were then sterilized by filtration through a syringe filter (Cellulose Acetate Syringe Filter, 0.45 µm, GE Healthcare Whatman).

### 4.5. Assessment of the Inhibitory Effect of Extract on Biofilm Formation—Extract Added at t_0_

#### 4.5.1. Formation of PAO1 Biofilms

Biofilms were developed in 24-well plates (Falcon, TC-treated, polystyrene). The bacterial suspension prepared in MBB (2X) was initially adjusted to 10^8^ CFU/mL followed by a serial dilution to 10^−6^ with the same medium. One milliliter of the 10^−6^ dilution (equivalent to 10^2^ CFU/mL) was introduced into each well. In order to test its effect on the biofilm, 1.0 mL of the algal extract (100.0 µg/mL) (sub-MIC Supplementary Materials S4) was added to each well, corresponding to a final concentration of 50.0 µg/mL. Wells containing 1.0 mL of SDW + 1.0 mL of un-inoculated MBB or 1.0 mL SDW + 1.0 mL inoculated MBB, were considered as sterility and biofilm growth controls, respectively. The plate was then incubated for 24 h at 37 °C. All assays were performed in triplicate.

#### 4.5.2. Screening of Algal Extracts for Their Effect on PAO1 Biofilm Formation and Growth—Crystal Violet Staining Method

The objective of this method was to quantify the total biomass of the biofilm (adhered cells + matrix) by crystal violet (CV) staining and consequently to evaluate the effect of the extract on the formation and proliferation of the biofilm [33]. The protocol adopted by Genovese et al., 2021 was followed with some modifications [56]. After overnight incubation, biofilms were washed twice with 2.0 mL of SDW to remove non-adherent planktonic cells. The plate was then air-dried for 1 h. To stain the adhered biomass, 2.0 mL of an aqueous 1% CV solution was added to the wells and consecutively incubated for 15 min at room temperature. In order to remove the excess stain, wells were rinsed twice with 2.0 mL of SDW followed by drying for 30 min before quantification. One milliliter of ethanol was finally added to extract bound stain and the inhibition percentage (IP_CV_) was calculated according to the following Formula (2):

(2) $${\mathrm{IP}}_{\mathrm{CV}}\left(\%\right)=\frac{{\mathrm{OD}}_{570\;\mathrm{nm}}\;\mathrm{of}\;\mathrm{biofilm}\;\mathrm{growth}\;\mathrm{control}-{\mathrm{OD}}_{570\;\mathrm{nm}}\;\mathrm{of}\;\mathrm{tested}\;\mathrm{extract}}{{\mathrm{OD}}_{570\;\mathrm{nm}}\;\mathrm{of}\;\mathrm{biofilm}\;\mathrm{growth}\;\mathrm{control}}\times 100$$

The absence of any interference between the extracts and CV staining was checked using blank wells (1.0 mL of extract + 1.0 mL cell-free MBB).

#### 4.5.3. Effect of the Potentially Active Extracts on the Number of Adhered Bacteria—CFU Counts Method

In this assay, the protocol developed by [8,26] was used with some modifications. After 24 h of incubation, wells were rinsed twice with 2.0 mL of SDW, and then the attached cells were scraped (for 1 min) with a sterile spatula into 1 mL of SDW. The recovered suspension was diluted by serial dilution (from 10^−1^ to 10^−6^) and 900 µL of each dilution was inoculated by inclusion in TSA agar plates. After 48 h of incubation at 37 ˚C, the numbers of CFU were counted by considering only plates with 15 to 300 CFU. The adhered biomass was then calculated and subjected to logarithmic transformation by Formula (3). The logarithmic reduction and the IP_CFU_ with respect to the corresponding untreated control were also calculated using Formulas (4) and (5).

(3) $$\log\mathrm{of}\;\mathrm{adhered}\;\mathrm{biomass}\;(\log\mathrm{CFU}/\mathrm{mL})=\log\;\frac{\mathrm{number}\;\mathrm{of}\;\mathrm{colonies}\;\left(\mathrm{CFU}\right)}{\mathrm{Dilution}\;\mathrm{factor}\times \mathrm{inoculated}\;\mathrm{volume}}$$

(4) logCFU/mL reduction=logCFU/mL for control−logCFU/mL for treated biofilm

(5) $${\mathrm{IP}}_{\mathrm{CFU}}\;\left(\%\right)=\frac{{\mathrm{Adhered}\;\mathrm{cells}}_{\;\mathrm{Control}}(\mathrm{CFU}/\mathrm{mL})-{\;\mathrm{Adhered}\;\mathrm{cells}}_{\;\mathrm{Sample}}(\mathrm{CFU}/\mathrm{mL})}{{\mathrm{Adhered}\;\mathrm{cells}\;}_{\mathrm{Control}}(\mathrm{CFU}/\mathrm{mL})}\times 100$$

#### 4.5.4. Phenotypic Observations by Epifluorescence Microscopy

The potential effect of extracts, added at t_0_, on PAO1 formed biofilm morphology and on bacterial cell organization was examined by epifluorescence microscopy (EM). For this analysis, *P. aeruginosa* biofilms were grown as described above but in a 6-well microplate (Falcon, TC-treated, polystyrene) and with a total volume of 6.0 mL (3.0 mL of PAO1 bacterial suspension prepared in MBB 2X (10^2^ CFU/mL) + 3.0 mL of tested extract or 3.0 mL of SDW for the control).

After 24 h of incubation at 37 °C, well content was carefully discarded and replaced by 6.0 mL of SDW. Live and damaged cells were differentiated by staining with 1.0 µL of Syto9 (5 mM, Invitrogen^TM^, ThermoFisher Scientific, Illkirch, France) and 1.0 µL of propidium iodide (1 mg/mL, Invitrogen^TM^, ThermoFisher Scientific), respectively.

Moreover, to examine the potential effect of extracts on the biofilm matrix, 1.0 mL of concanavalin A (ConA, tetramethylrhodamine conjugate, ThermoFisher Scientific) prepared at a concentration of 100.0 μg/mL in 0.1 M of sodium bicarbonate, was added to the well after its contents had been withdrawn. ConA is a lectin that exhibits an affinity for certain osidic residues, in particular for α-mannopyranosyl and α-glucopyranosyl residues. It is important to note that Strathman et al. [57] have proven that ConA may also bind to alginate, a component of *P.* *aeruginosa* biofilm matrix. Its conjugation to tetramethylrhodamine leads to the emission of orange-red visible fluorescence upon excitation with a green light. After 20 min of incubation in the dark at room temperature, wells were delicately rinsed twice with 1.0 mL of SDW. Just before proceeding to the microscopic observations, 6.0 mL of SDW, together with 1.0 μL of Syto9, were added. Microscopic observations were made with Zeiss—Axiotech microscope using a 20 X/0.50 (Zeiss, EC Plan-Neofluar) objective and equipped with an HXP 120 C light source. Images were acquired with a digital camera (Zeiss AxioCam ICm 1) and the set of photos was processed with ZEN software.

### 4.6. Effect of Selected Algal Extracts on PAO1 24 h-Old Biofilms—Extract Added at t_24 h_

Extracts for which the CV staining method revealed an effect on the biofilm formation (i.e., IP_CV_ > 50%) were subjected to an experiment to evaluate their potential impact on a 24-h-old biofilm. In this assay, 1.0 mL of algal extract solution (100.0 µg/mL) was added with 1.0 mL of MBB into wells of a 24-well plate in which a 24-h-old biofilm was developed as previously described. The plate was then re-incubated at 37 °C for 24 h. After incubation, wells were rinsed twice with 2.0 mL of SDW before the remaining biomass was quantified by the CV staining method.

The eradication percentage was calculated using the following Formula (6):

(6) $${\mathrm{EP}}_{\mathrm{CV}}\left(\%\right)=\frac{{\mathrm{OD}}_{570\;\mathrm{nm}}\;\mathrm{of}\;\mathrm{untreated}\;\mathrm{control}\;-{\;\mathrm{OD}}_{570\;\mathrm{nm}}\;\mathrm{of}\;\mathrm{tested}\;\mathrm{extract}}{{\mathrm{OD}}_{570\;\mathrm{nm}}\;\mathrm{of}\;\mathrm{untreated}\;\mathrm{control}}\times 100$$

The extract exhibiting an eradication percentage (EP_CV_) greater than 80% was also evaluated by the CFU counts method. In this case, both the adhered and the detached (planktonic) cells were quantified. To do this, before the wells were rinsed and scraped, 1.0 mL of the supernatant was withdrawn and submitted to serial dilution followed by inoculation in TSA agar for CFU quantification of planktonic cells. The adherent cells were quantified as described above. The number of CFU counted after 48 h of incubation at 37 °C was subjected to logarithmic transformation based on the above Formula (3).

### 4.7. Evaluation of the Synergistic Antibiofilm Activity of the Active Extract in Combination with Tobramycin or Colistin on 24 h-Old Treated Biofilms

The potential synergistic antibiofilm effect of the *U. lactuca* ethyl acetate (EA) active extract with tobramycin and colistin was evaluated on 24 h-old biofilms, previously treated with the EA extract or not, following the protocol developed by Furiga et al., 2016 with some modifications. Since the objective here was to detect a potential synergistic effect, the tested concentrations of antibiotics had to be lower than the concentration that would be fully effective in eradicating PAO1 biofilm, hence the choice of 2 and 16 µg/mL for tobramycin and colistin, respectively [41].

First, 1.0 mL of bacterial suspension (10^2^ CFU/mL) prepared in MBB (2X) was added into each well of a 24-well microplate, supplemented either with 1.0 mL of SDW (control, tobramycin, and colistin control) or with 1.0 mL of a solution of 100.0 µg/mL of EA extract (EA extract control and combination assays; final concentration 50.0 µg/mL). After 24 h of incubation at 37 °C, the supernatant was removed, and replaced by 1.0 mL of SDW (control) or 1.0 mL of tobramycin alone (tobramycin control; final concentration 2.0 µg/mL) or 1.0 mL of colistin alone (colistin control; colistin sodium methanesulfonate; final concentration 16.0 µg/mL) or 1.0 mL of EA extract (EA extract control; final concentration 50.0 µg/mL) or a solution of EA extract mixed with either tobramycin or colistin for the combination assays. The final concentrations of EA extract, tobramycin, and colistin were 50.0 µg/mL, 2.0 µg/mL and 16.0 µg/mL, respectively. MBB medium was then added to all wells (1.0 mL/well). For all conditions, the number of adherent cells after 48 h of incubation was quantified by the CFU counts method, as described above. Log reduction was then calculated using Formulas (3) and (4).

### 4.8. Analysis of the Chemical Composition of Extracts by GC–MS

The chemical composition of all extracts was analyzed first by GC–MS; extracts were prepared at a concentration of 2.5 mg/mL in the corresponding extraction solvent (cyclohexane, dichloromethane, ethyl acetate, or methanol). Analyses were performed using GC-MS system (TRACETM 1310—ThermoFisher Scientific) equipped with a Rtx-502.2 fused silica capillary column (30 m in length, 0.25 mm in diameter, 1.4 μm in film thickness). The column oven temperature was programmed as follows: initial temperature was 50 °C (for 2 min) then gradually increased to 150 °C (for 5 min) at a rate of 20 °C/min, and finally increased to 290 °C at a rate of 10 °C/min and maintained for 10 min. Ionization of the sample components was performed in electron impact mode (EI, 70 eV) with 220 °C as ion temperature. The injector and detector temperatures were 250 and 220 °C, respectively. Hydrogen was used as carrier gas at a flow rate of 1.0 mL/min. The injection volume of the prepared extract solution (2.5 mg/mL) was 5.0 μL. The total running time of the GC–MS system was 36 min. Finally, molecules were identified using Xcalibur software.

### 4.9. Statistical Analysis

All values were expressed as mean ± SD for three independent experiments. The student t-test was used to calculate the significance of the differences between the mean effects of the extract and those for the associated untreated control in the CFU counts method after checking equality of variances with Levene’s test (*p*-value < 0.05). Statistically significant values were defined as a *p*-value (* <0.05, ** <0.01 or *** <0.001). SPSS 22.0 software (SPSS, IBM Corporation, Armonk, NY, USA) was used in the statistical analysis.

## 5. Conclusions

In the present study, the screening of extracts derived from three algae for their antibiofilm activity against the pathogenic bacterium *P. aeruginosa* allowed two *U. lactuca* extracts (CH and EA extracts) to be selected as the most promising for valorization in this field. CH extract appears to impair microcolony growth, resulting in a significant reduction in the number of adherent cells, while an effect on the production and the degradation of the biofilm matrix has been suggested as a potential mode of action of EA extract. In light of these encouraging results, further experiments are envisaged to analyze the chemical composition of the two active extracts and isolate active components as pure molecules. The evaluation of the antibiofilm effect of these extracts on other pathogenic bacteria would identify a broad spectrum of activities. Overall, this study raises the possibility of extracting bioactive compounds from the green alga, *U. lactuca*, which can potentially be used alone or in combination with antibiotics in the treatment of biofilm-related infections.

## Figures and Tables

**Table 1:** Characteristics of extracts according to the extraction solvents.

Seaweed Species		CH P’: 0.2	DCM P’: 3.1	EA P’: 4.4	MeOH P’: 5.1
Green alga *U. lactuca*	N° of repetitions	×1	×2	× 2	×4
	Color	Pale yellow	Dark green	Dark green	Dark green
	Yield (*w*/*w*%)	0.2	0.3	0.1	12.1
Brown alga *S. scoparium*	N° of repetitions	×2	×3	×3	×3
	Color	Dark yellow	Dark green	Dark green	Green
	Yield (*w*/*w*%)	0.2	0.2	0.5	1.4
Red alga *P. capillacea*	N° of repetitions	×2	×3	×3	×4
	Color	Dark yellow	Dark green	Dark green	Dark green
	Yield (*w*/*w*%)	0.4	0.8	0.9	7.3

P’: Polarity index. CH: cyclohexane, DCM: dichloromethane, EA: ethyl acetate, MeOH: methanol.

**Table 2:** Comparison of the antibiofilm activity of selected algal extracts (50.0 µg/mL) using the crystal violet staining method and the numeration of adherent bacteria (CFU counts) method.

Seaweed Species	Nature of the Extract	CV Method	CFU Method	
		IP_CV_ (%)	IP_CFU_ (%)	Log Reduction in Relation to Untreated Control
Green alga (*U. lactuca*)	CH	69.4 ± 13.6	67.2 ± 17.2	0.5 ± 0.1 **
	DCM	52.9 ± 9.2	NA	0
	EA	84.0 ± 9.6	44.3 ± 16.5	0.2 ± 0.2 ^NS^
Brown alga (*S. scoparium*)	DCM	75.2 ± 15.4	28.1 ± 24.1	0.1 ± 0.1 ^NS^
	EA	64.8 ± 3.6	NA	0

Extracts were added at t_0_. Results are expressed as means of inhibition percentage (IP_CV_ and IP_CFU_) ± SD and log reduction in comparison with the related untreated control (log reduction (log CFU/mL) ± SD) for the CFU counts method, from three independent experiments. Statistically significant difference (**, *p*-value < 0.01) between the extract and the related untreated control is indicated. ^NS^: not significant, NA: not active (IP < 10%).

**Table 3:** Compounds identified in the extracts derived from the green alga *U. lactuca*, the brown alga *S. scoparium*, and the red alga *P. capilllacea*. MF: Molecular formula. MW: Molecular weight. RT: Retention time.

Identified Molecules	MF	MW (g/mol)	RT (min)											
			*U. lactuca*				*S. scoparium*				*P. capillacea*			
			CH	DCM	EA	MeOH	CH	DCM	EA	MeOH	CH	DCM	EA	MeOH
2,4-Dithiapentane	C_3_H_8_S_2_	108						7.37						
2,4-Di-tert-butylphenol/2,5-bis(1,1-dimethylethyl)-phenol	C_14_H_22_O	206		18.81	18.96	18.55		18.76	19.36	18.45		19.04	19.24	18.54
Heptadecane	C_17_H_36_	240				20.33	19.72	20.24		20.04	19.88	20.5	20.68	
3,5-bis(1,1-dimethylethyl)-4-hydroxy-methyl ester benzenpropanoic acid	C_18_H_28_O_3_	292				23.5		24.5						
2,6-bis(1,1-dimethylethyl)-4-(1-methyl-1-phenylethyl)-phenol	C_23_H_32_O	324	25.66	26.08	26.2			26.03	26.59		25.67	26.29	26.41	
2,4-Bis(dimethylbenzyl)-6-t-butylphenol	C_28_H_34_O	386	32.49	33.48	33.7		32.22	33.37	34.77		32.52	34.03	34.27	
1-ethynyl-4-methyl benzene	C_9_H_8_	116		9.67								9.93		
6,10,14-trimethyl-2-pentadecanone	C_18_H_36_O	268		22.59										
Hexadecanoic acid methyl ester	C_17_H_34_O_2_	270	23.08	23.48							23.09		23.8	
Decane	C_10_H_22_	142	7.37											
Nonanal	C_9_H_18_O	142	9.39											
Isopropyl myristate	C_17_H_34_O_2_	270	21.83											
Tetratriacontane	C_34_H_70_	478	26.29											
Hexadecanoic acid ethyl ester	C_18_H_36_O_2_	284							24.61				24.46	
2,4-bis(1-methyl-1-phenylethyl) phenol	C_24_H_26_O	330	33.12		34.5				35.58				35.03	
1-ethoxy-2-propanol	C_5_H_12_O_2_	104											5.79	
4-hydroxy-4-methyl-2-pentanone	C_6_H_12_O_2_	116											6.99	
1-Ethoxypropane-2-yl-acetate	C_7_H_14_O_3_	146											7.68	
4-(2,6,6-trimethyl-2-cyclohexen-1-yl)-3-buten-2-one	C_13_H_20_O	192											18.12	
5,6,7,7a-tetrahydro-4,4,7a-trimethyl-2(4H)-benzofuranone	C_11_H_16_O_2_	180											20.93	
Methyl tetradecanoate	C_15_H_30_O_2_	242											21.52	
6,10,14-trimethyl-2-pentadecanone	C_18_H_36_O	268											22.93	
Dibutyl phtalate	C_16_H_22_O_4_	278											25.28	
phytol	C_20_H_40_O	296											25.81	
3,7,11,15-tetramethylacétate-2-hexadecen-1-ol	C_22_H_42_O_2_	338											26.73	

## Supplementary Materials

The following are available online at https://www.mdpi.com/article/10.3390/md20020092/s1, Figure S1: Map showing the area where seaweed samples were collected, Figure S2: Planktonic growth kinetics of PAO1 in MHB, MBB and in presence of EA, Table S3: Evaluation of the potential bactericidal activity of CH and EA extracts on PAO1. Figure S5: Chromatograms of extracts derived from the green alga *U. lactuca*.
