# Supplementary material for: Seaweed Extracts: A Promising Source of Antibiofilm Agents with Distinct Mechanisms of Action against Pseudomonas aeruginosa
Source: Mar Drugs. 2022 Jan 21;20(2):92. doi: 10.3390/md20020092 (PMC8877608; doi:10.3390/md20020092)
Supplement: Supplementary file 1 [file marinedrugs-20-00092-s001.zip › marinedrugs-1543018-supplementary.pdf]

marine drugs

MDPI

Article – Supplementary materials

Seaweed extracts: A promising source of antibiofilm agents with distinct mechanisms of action against *Pseudomonas aeruginosa*

Maya Rima [1,2], Jeanne Trognon [1], Laure Latapie [1], Asma Chbani [2,3], Christine Roques [1,4,*], Fatima El Garah [1,*]

[1] Laboratoire de Génie Chimique, Université de Toulouse, CNRS, INPT, UPS, Toulouse, France
[2] Laboratory of applied biotechnology, AZM Center for Research in Biotechnology and its Applications, Doctoral School of Science and Technology, Lebanese University, El Mittein Street, Tripoli, Lebanon.
[3] Faculty of Public Health III, Lebanese University, Tripoli, Lebanon
[4] Bacteriology-Hygiene Department, Centre Hospitalier Universitaire, Toulouse, Hôpital Purpan, Toulouse, France
* Correspondence: Dr Fatima El Garah, Tel: +33 562256855, e-mail: fatima.el-garah@univ-tlse3.fr, ORCiD ID : 0000-0001-7217-5879; Professor Christine Roques, Tel: +33 562256860, e-mail: christine.roques@univ-tlse3.fr

S1: Collection of algal materials

Seaweed samples belonging to three different groups (green alga *Ulva lactuca*, brown alga *Stypocaulon scoparium*, and red alga *Pterocladiella capillacea*) were manually collected in the Mediterranean Sea, from the northern Lebanese coast, particularly from El Mina in Tripoli in September 2019 (Figure S1).

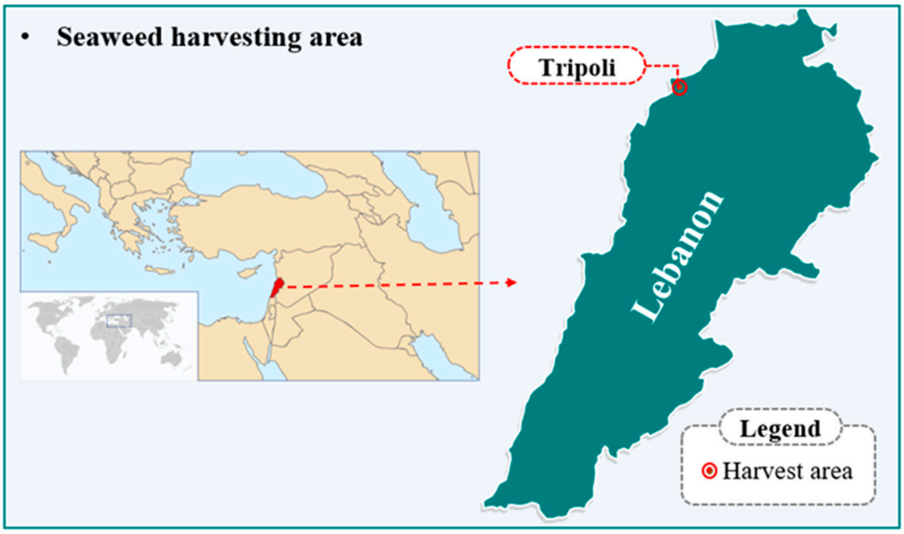

Figure S1. Map showing the area where seaweed samples were collected.

S2: PAO1 planktonic growth kinetics in the rich medium MHB, in the low-nutritive medium MBB and in presence of EA-Extract – optical density measurement

PAO1 growth kinetics curves in MHB, MBB, and in MBB in the presence of EA extract were also performed. Briefly, 100 µl of tested media (MHB and MBB 2X) were introduced into the wells of a sterile 96-well microtiter plate (Falcon, TC-treated, polystyrene) supplemented with 100 µl of sterile distilled water. In order to evaluate the potential effect of EA extract on the PAO1 growth curve, 100 µl of EA extract stock solution (100.0 µg/ml) were added to 100 µl of MBB. The bacterial suspension used in this assay was prepared in sterile distilled water and adjusted to an optical density of 0.150 at 640 nm, corresponding to a concentration of 10^8 CFU/ml followed by dilution (1:10) to achieve a concentration of 10^7 CFU/ml. Then, the microtiter-plate was inoculated using a manual multipoint inoculator. Note that wells in the last column were used as sterility controls (100 µl of sterile distilled

water + 100 µl of tested media). The plate was incubated at 37°C for 24h in a microplate spectrophotometer (Multiskan™ GO, Thermo Fisher Scientific, Waltham, USA) under continuous agitation. The optical density measurement was carried out at 640 nm every one hour. The measured values were plotted as a function of time. Results are expressed as means ± SD (OD_{640nm}) of two independent assays (Figure S2).

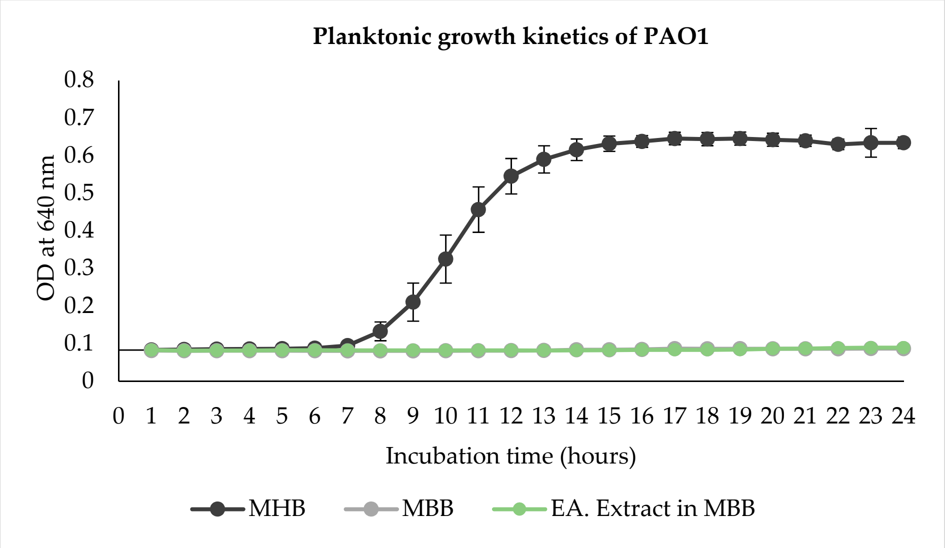

Figure S2. Planktonic growth kinetics of PAO1 in MHB, MBB and in presence of EA. Extract (50.0 µg/ml) originated from the green alga *U. lactuca*. Results are expressed as means ± SD of the optical density measured at 640 nm of two independent experiments. EA is ethyl acetate extract. MHB and MBB are Mueller-Hinton broth and modified biofilm broth, respectively.

S3: Checking the potential bactericidal activity of CH and EA extracts derived from the green alga *U. lactuca* – CFU counts method

In order to exclude the potential bactericidal effect of the two active extracts (CH and EA extracts derived from the green alga *U. lactuca*) at the tested concentration (50.0 µg/ml), their effect on PAO1 planktonic cells was assessed. The protocol developed by Feuillolay et al., 2016 was used in this assay. Briefly, 5.0 ml of PAO1 bacterial suspension (10^5 or 10^2 CFU/ml) prepared in MBB (2X) medium and supplemented with 5.0 ml of sterile distilled water were incubated for 24 hours at 37˚C. Water was replaced by 5.0 ml of extracts (CH or EA extracts) in the sample tubes. The potential bactericidal activity of extracts was determined on both suspension (10^5 and 10^2 CFU/ml). Tubes were maintained under agitation (100 rpm) in an orbital shaker (MaxQ 4000, Thermo Fisher Scientific, Waltham, USA). The number of planktonic cells was monitored after 24 hours of incubation at 37°C by CFU counts. Prior to their quantification, samples were homogenized then 1.0 ml was taken and serially diluted (10^{-1} to 10^{-6}). 900 µl of each dilution were inoculated by inclusion in TSA agar plates and overnight incubated at 37˚C for cell quantification. Assays were performed in duplicate. Results expressed as ratio (log CFU/ml for sample / log CFU/ml for control) are presented in Table S3.

Table S3. Evaluation of the potential bactericidal activity of CH and EA extracts (50.0 µg/ml) derived from the green alga *U. lactuca* on PAO1 (10^5 CFU/ml or 10^2 CFU/ml). The number of planktonic cells was measured after 24 hours of incubation at 37°C under agitation. Results are expressed as means of ratio (log CFU/ml for sample/ log CFU/ml for control) ± SD from two independent experiments. CH and EA are cyclohexane and ethyl acetate extracts, respectively.

Initial bacterial suspension	CH extract	EA extract
**10^5 CFU/ml**	1.03 ± 0.01	1.01 ± 0.01
**10^2 CFU/ml**	1.00 ± 0.02	1.00 ± 0.01

Feuillolay, C., Pecastaings, S., Le Gac, C., Fiorini-Puybaret, C., Luc, J., Joulia, P., & Roques, C. (2016). A Myrtus communis extract enriched in myrtucummulones and ursolic acid reduces resistance of Propionibacterium acnes biofilms to antibiotics used in acne vulgaris. *Phytomedicine* , *23*, 307-315.

S4: Effect of extracts on PAO1 planktonic growth – MIC determination

The antibacterial activity was evaluated in order to determine the appropriate concentration of the extracts to be used in the antibiofilm activity assays (sub-MIC) in a way that they did not present classical bacteriostatic/bactericidal effects since we were looking for an effect on the biofilm formation. The MIC of each extract against *P. aeruginosa* was determined using the broth microdilution method, according to the guidelines of CA-SFM/EUCAST 2020. Briefly, 100.0 µl samples of algal extract solution (100.0 µg/ml) were introduced into the wells of the first column of a sterile 96-well microtiter plate (Falcon, TC-treated, polystyrene) and subjected to 2-fold serial dilutions with Mueller-Hinton broth (MHB) (100 µl/well) to achieve final concentrations ranging from 50.0 to 0.098 µg/ml. The bacterial suspension used in this assay was prepared in SDW and adjusted to an optical density of 0.150 at 640 nm, corresponding to a concentration of about 10^8 CFU/ml. This suspension was then subjected to a 2-fold dilution in SDW prior to the inoculation of the microtiter-plate using a manual multipoint inoculator (1.0 µl), in order to obtain a final concentration of 5 x 10^5 CFU/ml. Note that wells in the last column were used as sterility controls (SDW + MHB). The previous column was dedicated to growth control (SDW + MHB + inoculum). After incubation at 37˚C for 24 h, the MIC, defined as the lowest concentration of the tested extract that could prevent visible bacterial growth, was determined. Assays were performed in duplicate. According to the results, no antibacterial activity was demonstrated at the highest concentration tested (50.0 µg/ml) for any of the extracts. Therefore, the concentration adopted for the antibiofilm activity assays was 50.0 µg/ml for all extracts.

S5: Chromatograms of extracts derived from the green alga *U. lactuca* – GC/MS

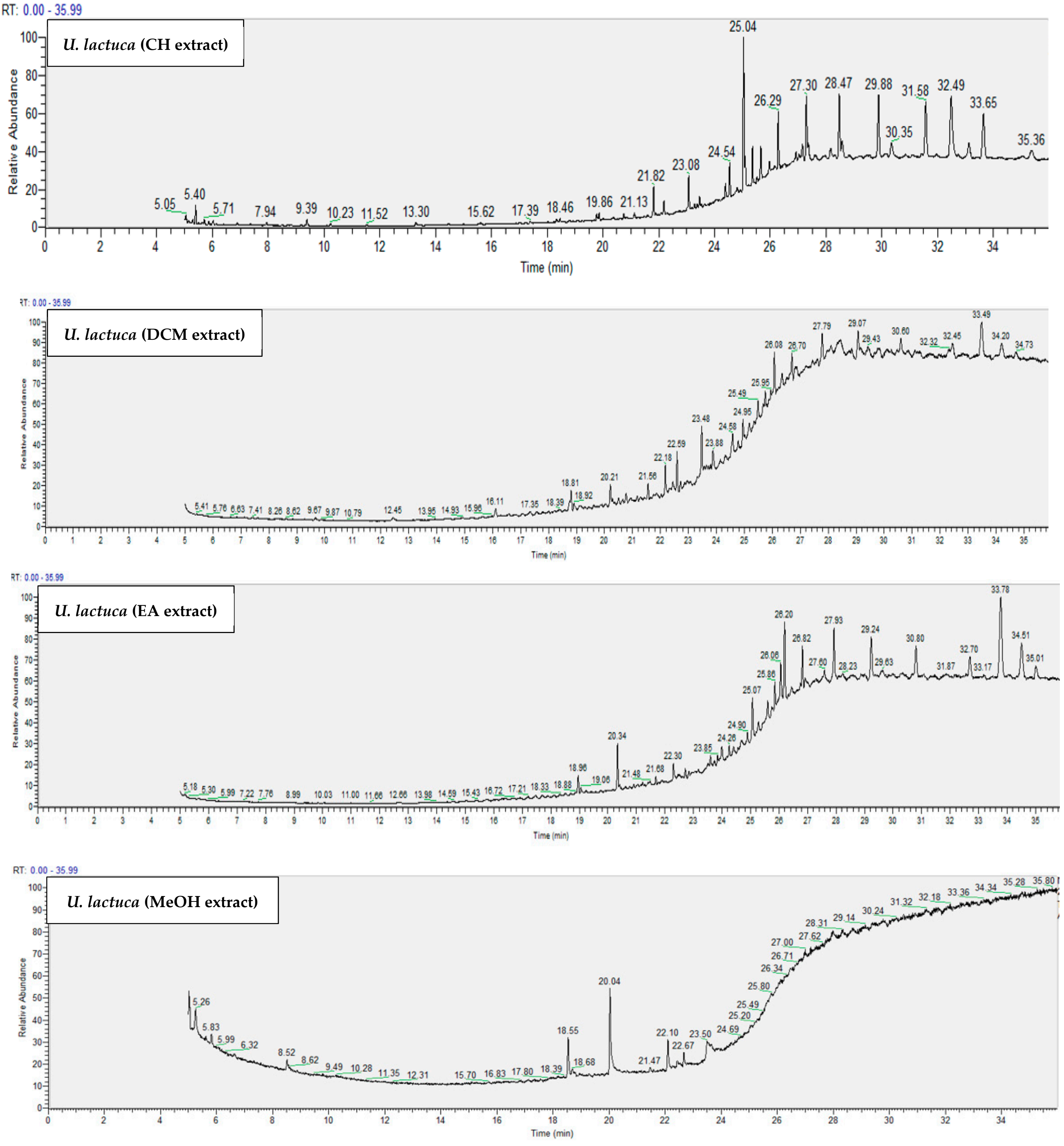

Figure S5. Chromatograms of extracts derived from the green alga *U. lactuca*.
